# Regional differences regarding the occurrence of falls and associated factors in two populations of Brazilian longevous people

**DOI:** 10.1186/s12877-022-03630-2

**Published:** 2022-12-02

**Authors:** Jaíza M. M. Silva, Javanna Lacerda Gomes da Silva Freitas, Júlia Cristina Leite Nóbrega, Juliana Barbosa Medeiros, Raisa Fernandes Mariz Simões, Ricardo Olinda, Jair Lício de Ferreira Santos, Yeda Aparecida de Oliveira Duarte, Mayana Zatz, David Matheson, Silvana Santos, Tarciana Nobre Menezes

**Affiliations:** 1 Public Health Program, Universidade Estadual da Paraíba (UEPB), Campina Grande, Brazil; 2 Department of Statistics, Universidade Estadual da Paraíba (UEPB), Campina Grande, Brazil; 3 Department of Social Medicine, Universidade de São Paulo (USP), Campina Grande, Brazil; 4 Department of Medical Surgical Nursing, School of Nursing, Universidade de São Paulo, São Paulo, Brazil; 5 Human Genome Studies Center, Universidade de São Paulo (USP), São Paulo, Brazil; 6 Faculty of Education, Health and Wellbeing, University of Wolverhampton, Wolverhampton, UK; 7 Department of Biology, Universidade Estadual da Paraíba (UEPB), Campina Grande, Brazil; 8 Department of Physical Therapy, Universidade Estadual da Paraíba (UEPB), Campina Grande, Brazil

**Keywords:** Postural balance, Psychomotor performance, Falls, Aged 80 years or older

## Abstract

**Background:**

Few studies have explored regional asymmetries and their implications for health policies regarding episodes of falls among the population of ≥80 years old in continental and developing countries like Brazil with deep inequalities and sociocultural differences.

**Objective:**

To evaluate the occurrence of falls and their association with functional capacity and nutritional status in the longest oldest-old living in two municipalities in the Northeast and Southeast of Brazil.

**Methods:**

This is a cross-sectional study, with primary data collection in which were included in the research seniors aged 80 years or more, of both sexes, belonging to two Brazilian municipalities of discrepant socioeconomic aspects. The dependent variable was the occurrence of falls in the last year. The independent variables were grouped into demographic aspects, functional capacity and nutritional status. To identify variables that contribute to the occurrence of falls, the multiple logistic regression model, adopts a significance level of 5%.

**Results:**

The sample was composed of 415 oldest-old adults. From the total, 32.3% reported having fallen in the last year, 24.7% in Brejo dos Santos and 37.8% in São Paulo. Among the former population, the mean value of walking speed for those who had falls was 0.27 m/s and for those who had no occurrence of falls was 0.33 m/s; and, among the seniors from São Paulo, the mean values were 0.51 m/s and 0.58 m/s, respectively. Significant correlations between walking speed and falls were verified for both populations, showing that the lower the walking speed, the higher the predisposition to falls. In the final regression model, the occurrence of falls was associated with moderate balance (OR = 5.28; CI: 1.11–25.18) among the longevous people Brejo dos Santos and with very poor functional performance (OR = 16.09; CI:1.46–177.06) among those from São Paulo.

**Conclusion:**

The results pointed out a lower prevalence of falls in longevous people from Brejo dos Santos than in those from São Paulo and differences regarding the associated factors, showing heterogeneity between the two populations; indicating the need for public policies and effective programmes aimed at preventing falls based on the maintenance or increase of functional capacity.

## Background

The demographic and epidemiological transition has contributed to a growing increase in the proportion of older people in the world, whose numbers are estimated to reach 1.4 billion by 2030 and 2.1 billion by 2050, and could rise to 3.2 billion in 2100 [1]. Longevity is a multifactorial and complex characteristic, with the contribution of genetic and environmental factors [2, 3]. Differences in socioeconomic, demographic, cultural and lifestyle conditions can influence the life expectancy of the population [4, 5]. In centenarians from Sardinia in Italy, for example, a low female-to-male ratio and an association between longevity and consanguinity was observed [6–10]. A similar pattern was noticed among those 80-years-old or older in Brejo dos Santos, in Paraíba, where there is a high rate of consanguineous marriages [11]. In this population from the Northeast of Brazil, differences were also described in relation to factors associated with functional capacity [12, 13] and health-related quality of life [14] when compared to São Paulo, in the Southeast of the country, suggesting that there were regional differences in the health indicators of oldest-old populations in Brazil.

The physiological alterations inherent to the human ageing process, the presence of chronic diseases and the effects caused by excessive intake of medication, as well as the decrease in functional capacity and nutritional status, were associated with episodes of falling [15]. A fall is an unexpected event in which the participant comes to rest on the ground, floor, or a lower level [16]. Falls affect 50% of adults over the age of 80 [15]. Many falls result in fractures as well as soft tissue injuries, long-standing pain, functional impairments, reduced quality of life, increased mortality, and excess in healthcare costs [17]. Intrinsic risk factors of falling include characteristics of the individual such as age, functional abilities, chronic diseases and gait disturbances [18]; while extrinsic or environmental risk factors refer to fall hazards in and around the home such as poorly fitting footwear, slippery floor, lack of stair railings, unstable furniture, and poor lighting [15]. Structured fall-preventive programmes, especially in high-risk groups, are beneficial in reducing both the number of fallers and the number of falls among older adults [17].

Since 1994, based on the Brazilian National Policy for the Old People, national and state governments have implemented policies and actions to ensure seniors’ social rights, autonomy, integration, and effective participation in society [19]. These policies have also pointed out the importance of maintaining functional capacity, the early detection of non-communicable diseases, and the use of protocols for fall risk situations [20]. Despite the improvement in public policies and access to health services for the older people, family is generally their main source of social support [21]; and it is therefore important to promote public policies that can provide ways to support families that are caring for older adults [22, 23].

Thus, it is clear that actions to prevent falls need to consider the context in which these oldest-old live, as well as the multi-dimensional character of the causes of these falls. As far as is known, only a few studies have explored regional asymmetries and their implications for health policies regarding episodes of falls in continental and developing countries like Brazil which have deep inequalities and sociocultural differences. In this study, the objective was to evaluate the occurrence of falls and their association with functional capacity and nutritional status in seniors aged 80 years or more, living in a poor and rural municipality in the Brazilian Northeast and in São Paulo, one of the largest urban centres in Latin America. In Brazil, there are few studies that have evaluated these factors in the population of oldest-old aged 80 years or more, considering that in this age group there occur more frequent episodes of falls, which may have disabilities and dependencies as consequences; hence, becoming a relevant social, economic and health issue.

## Methods

The Health, Wellbeing and Ageing (Saúde, Bem-Estar e Envelhecimento – SABE”) study aims to assess the living and health conditions of older people in order to project the social and health needs of the oldest-old population [24]. In Brazil, this study was initiated in São Paulo (SABE-SP) and later extended to rural and consanguineous populations in Northeast Brazil, with research being carried out in the town of Brejo dos Santos in Paraíba (SABE-PB) [12–14].

### Population and procedures

Data collection in Brejo dos Santos/PB, a municipality located in the micro-region of Catolé do Rocha, took place from May to September 2017. In 2010, according to the census conducted by the Brazilian Geographical and Statistical Institute (Instituto Brasileiro de Geografia e Estatística – IBGE), the town of Brejo dos Santos had 6198 inhabitants, of whom 14.1% were older adults, aged 60 years or more [25]. The criterion for the selection of the municipality was due to the high prevalence of consanguinity and the existing partnership between the health secretariat and researchers from the Paraíba State University (UEPB).

Data collection in the municipality of São Paulo occurred as part of a cohort of the SABE Study carried out between March and June 2016. In 2010, according to the IBGE census, the city of São Paulo had 11,253,632 inhabitants, of which 11.9% were seniors aged 60 years or more [25]. In Brazil, the SABE Study was developed in the city of São Paulo, which, although it was not the city with the highest proportion of older people in the country, represented, and still represents, the largest absolute number of them as well the older population with the greatest diversity, as a result of immigration and internal relocation [26].

Altogether, 188 seniors aged 80 years or more from the rural city of Brejo dos Santos/PB were located and referred by health workers. Of these 188, 179 agreed to participate in this research. With a probabilistic and representative sample of the population of São Paulo, 238 oldest-old adults in the urban centre were included in this study. In both populations, face-to-face interviews using the SABE form were conducted in the homes of the seniors by previously trained researchers, after signing the informed consent form.

### Study variables

#### Dependent variable

The occurrence of falls in the last 12 months before the interview was considered as a dependent variable. This information was obtained through the following question: “Have you had a fall in the last 12 months (last year)?”. The participants were divided into two categories: with the occurrence of falls (those who answered positively) and without the occurrence of falls (those who answered negatively).

#### Independent variables

The independent variables were grouped into demographic aspects, such as age group (80–85 years; 90 years or more), gender, functional capacity and nutritional status. The functional capacity was verified through the gait speed test, balance test, lower limb strength test, functional performance test and through the change in mobility.

For the walking speed test, the participant was instructed to walk at his/her usual speed for three metres in a straight line and time. Two measurements were made, and the shorter execution time of the test was used for this study. The average speed was calculated by dividing the distance by the time the individual reaches to complete this distance (m/s).

Balance was verified by means of three separate measures as proposed by Guranilk and colleagues [27]. The participants were asked to perform each one of the following measures: 1) maintain balance with both feet together (side by side) for 10 seconds; 2) maintain balance with one foot slightly in front of the other; 3) maintain balance with the heel of one foot directly in front of the other foot.

The first two measures were considered successful when the participants were able to remain 10 seconds in the required position. In instances where the senior could not perform the first measurement, they were not to perform the second and third ones. For the third measure, a score of three points was given for staying in this position for 3 to 9 seconds and a maximum score of four points was given if the oldest-old participant could stay in this same position for 10 seconds. Participants who presented clinical weakness or who did not have the physical or cognitive capacity to perform the test were excluded.

The participant was classified according to the number of measurements performed as follows [27]:

**Table:** 

Balance	Criterion
Very poor balance	Could only perform the first measurement
Poor balance	Could perform two measurements
Moderate balance	Could perform three measurements with scoring of three points
Good balance	Could perform three measurements with a maximum score of four points.

To evaluate the strength of the lower limbs, the test of getting up from a chair was performed [27]. To perform the test, the participants were asked if they felt safe to get up from the chair only once, without using their own arms. If they answered yes, they were invited to perform the test. After successful completion of the task, they were asked to cross their arms over their chest and stand up and sit down five times as fast as possible. These movements were timed from the initial sitting position to the final position at the end of the fifth position.

The test was performed successfully when performed in less than 60 seconds. The participants who performed the test between > 16.7 and ≤ 60 seconds were classified with “very poor strength”; between ≥13.7 and ≤ 16.6 seconds with “poor strength”; between ≥11.2 and ≤ 13.6 seconds with “moderate strength”; and those who performed the test in a time ≤ 11.1 seconds were classified with “good strength” [27]. Seniors with clinical weaknesses or those who did not have the capacity to understand the test were excluded.

The functional performance was evaluated by means of the Short Physical Performance Battery (SPPB), which consists of a battery of tests that evaluates balance, gait speed and lower limb strength [27]. The sum of the points in each of these parameters results in a score, which ranges from 0 (incapable or worse performance) to 12 (better performance). Seniors with incapacity or very poor performance obtain 0 to 3 points; 4 to 6 points, for low performance; 7 to 9 points, for moderate performance and 10 to 12 points, for good performance.

Alteration in mobility was evaluated through self-reporting of difficulty in performing various actions and movements, including walking a block along a street; sitting for two hours; getting up from a chair; bending down, kneeling or squatting; lifting or carrying weights greater than 5 kilogrammes (kg). The categories used were the presence of altered mobility, whether the participant answered “yes” or “cannot do” to the questions related to the activities mentioned, and unaltered mobility when the participant did not report difficulty and answered “no” or “can do”.

Nutritional status was assessed by means of the Body Mass Index (BMI) and Calf Circumference (CC). The weight (kg) was measured with a portable digital scale (TANITA UM080) and the height with a stadiometer, following the technique proposed by Gordon and colleagues [28]. The BMI was a result of the ratio between weight and height squared (kg/m2). Seniors classified according to the proposal of the Nutrition Screening Initiative (NSI) [29]: underweight (BMI ≤ 22 kg/m2), appropriate weight (22 < BMI < 27 kg/m2), overweight (BMI ≥ 27 kg/m2). The Calf Circumference (CC) was measured using the technique proposed by the Brazilian Association of Nutrology [30] using an inextensible measuring tape. For the classification of the CC, the classification proposed by the WHO [31] was considered malnutrition CC < 31 cm and eutrophy CC ≥ 31 cm.

The losses consisted of the difficulty of the participants to perform the physical tests, as well as the impossibility of measuring certain anthropometric variables, considering the difficulties to stand up and the physical disabilities. Due to the physical and cognitive limitations of the participants to perform the tests proposed in this work, as well as the impossibility of measuring certain anthropometric variables, the final sample for statistical analysis excluded losses. As shown in Table 1, it was possible to collect the answers from 113 participants from Brejo dos Santos and 171 from São Paulo for balance assessment; 133 individuals were unable to answer. Similarly, it was not possible to collect answers from 164 seniors for lower limb strength assessment, 134 for functional performance assessment through SPPB, and 17 for assessment of mobility alteration. Regarding the nutritional status, the body mass index was not evaluated for 131 participants, and the calf circumference was for 31 seniors.

**Table 1:** Distribution of the oldest-old populations, according to the occurrence of falls, gender, age group, functional capacity and nutritional status. Brejo dos Santos/PB and São Paulo/SP, Brazil, 2017

Variables	Occurrence of falls					
	Brejo dos Santos			São Paulo		
	(***N*** = 179)			(***N*** = 238)		
	Yes	No		Yes	No	
	n(%)	n(%)	***Total***	n(%)	n(%)	***Total***
**Demographic aspects**						
Gender						
Female	28(63.6)	70(52.2)	*98 (55.1)*	66(73.3)	102(69.4)	*168(70.9)*
Male	16(36.4)	64(47.8)	*80(44.9)*	24(26.7)	45(30.6)	*69(29.1)*
**Total**	44 (100)	134(100)	*178(100)*	90(100)	147(100)	*237(100)*
Age group						
80–89 years	35(79.5)	105(78.4)	*140(78.6)*	63(70.0)	110(74.8)	*173(73.0)*
90 years and over	9(20.5)	29(21.6)	*38(21.4)*	27(30.0)	37(25.2)	*64(27.0)*
	44(100)	134(100)	*178(100)*	90 (100)	147 (100)	*237(100)*
**Functional capacity**						
Balance						
Very poor	6(21.4)	10(11.8)	*16(14.2)*	14(21.2)	18(17.1)	*32(18.7)*
Poor	11(39.3)	21(24.7)	*32(28.3)*	12(18.2)	15(14.3)	*27(15.8)*
Moderate	4(14.3)	5(5.9)	*9(8.0)*	16(24.2)	15(14.3)	*31(18.1)*
Good	7(25.0)	49(57.6)	*56(49.6)*	24(36.4)	57(54.3)	*81(47.4)*
**Total**	28(100)	85(100)	*113(100)*	66(100)	105(100)	*171(100)*
Lower limb strength						
Very poor	14(50.0)	30(35.3)	*44(38.9)*	34(68.0)	61(67.8)	*95(67.9)*
Poor	7(25.0)	18(21.2)	*25(22.1)*	7(14.0)	17(18.9)	*24(17.1)*
Moderate	4(14.3)	15(17.6)	*19(16.8)*	6(12.0)	4(4.4)	*10(7.1)*
Good	3(10.7)	22(25.9)	*25(22.1)*	3(6.0)	8(8.9)	*11(7.9)*
**Total**	28(100)	85(100)	*113(100)*	50(100)	90(100)	*140(100)*
Functional performance						
Unable/very poor	7(25.0)	9(10.6)	*16(14.2)*	17(26.2)	14(13.3)	*31(18.2)*
Low	14(50.0)	37(43.5)	*51(45.1)*	22(33.8)	37(35.2)	*59(34.7)*
Moderate	6(21.4)	28(32.9)	*34(30.1)*	22(33.8)	42(40.0)	*64(37.6)*
Good	1(3.6)	11(12.9)	*12(10.6)*	4(6.2)	12(11.4)	*16(9.4)*
**Total**	28(100)	85(100)	*113(100)*	65(100)	105(100)	*170(100)*
Mobility impaired						
Yes	40(93.0)	112(86.8)	*152(88.4)*	78(88.6)	112(80.0)	*190(83.3)*
No	3(7.0)	17(13.2)	*20(11.6)*	10(11.4)	28(20.0)	*38(16.7)*
**Total**	43(100)	129(100)	*172(100)*	88(100)	140(100)	*228(100)*
**Nutritional Status**						
Body Mass Index						
Underweight	13(35.1)	27(25.7)	*40(28.2)*	11(14.9)	19(17.6)	*30(20.8)*
Adequate	15(40.5)	53(50.5)	*68(47.9)*	38(51.4)	48(44.4)	*48(33.3)*
Overweight	9(24.4)	25(23.8)	*34(23.9)*	25(33.8)	41(38.0)	*66(45.8)*
**Total**	37(100)	105(100)	*142(100)*	36(100)	108(100)	*144(100)*
Calf Circumference						
Malnutrition	24(57.1)	77(60.6)	*101(59.8)*	16(18.6)	33(25.2)	*49(22.6)*
Eutrophic	18(42.9)	50(39.4)	*68(40.2)*	70(81.4)	98(74.8)	*168(77.4)*
**Total**	42(100)	127(100)	*169(100)*	86(100)	131(100)	*217(100)*

### Data processing and analysis

Data were tabulated in the Epidata 3.1 programme in double entry and, soon after, transferred to a Microsoft Excel 2010 spreadsheet. After descriptive and analytical analysis, the multiple logistic regression model was adjusted with all variables and used as a measure of association with the odds ratio (OR) and 95% confidence intervals (CI). The adjustment variables that showed a significance of at least 20% (*p* ≤ 0.20) in the initial multiple logistic regression model were included in the final model. The correlation between the continuous variable gait speed and the occurrence of falls was verified separately, by means of logistic regression.

### Ethical issues

The larger projects, of which this study is part, were approved by the Research Ethics Committee of the Paraíba State University for the research in Brejo dos Santos (CAEE: 2.067.618) and by the Research Ethics Committee of the Public Health School of the University of São Paulo for the research conducted in the municipality of São Paulo (CAEE: 2044) and are in accordance with the ethical aspects involving research with human beings. After receiving verbal and written explanations about the study, the seniors who agreed to participate were asked to sign the Informed Consent Form.

## Results

We interviewed 417 oldest-old adults, with a mean age of 86.25 years (SD = 5.03), whose ages ranged from 80 to 102 years. Of these, 179 belong to the municipality of Brejo dos Santos and 238 to the municipality of São Paulo. Of the 417 seniors, two did not answer the questionnaire about the occurrence of falls in the last 12 months. Thus, among the 415 seniors assessed, 32.3% reported an occurrence of falls in the past year, being 24.7% in Brejo dos Santos and 37.8% in São Paulo. Among the participants from Brejo dos Santos/ PB, the mean value of walking speed for those who had fallen was 0.27 m/s and for those who had no occurrence of falls was 0.33 m/s. Among the seniors from São Paulo/ SP, the mean value of walking speed is 0.51 m/s for those who fell and 0.58 m/s for those who did not fall.

Table 1 shows the distribution of the senior residents in Brejo dos Santos and São Paulo, according to the occurrence of falls, gender, age group, functional capacity and nutritional status. Among the oldest-old people living in both cities who presented with the occurrence of falls, most of them were female, in the age group from 80 to 89 years old, with very poor lower limb strength, low functional performance, altered mobility and appropriate body weight. There were differences regarding balance and CC, whereby most of the seniors from Brejo dos Santos were classified with bad balance and malnutrition, and the seniors from São Paulo with good balance and eutrophic.

**Fig. 1:**
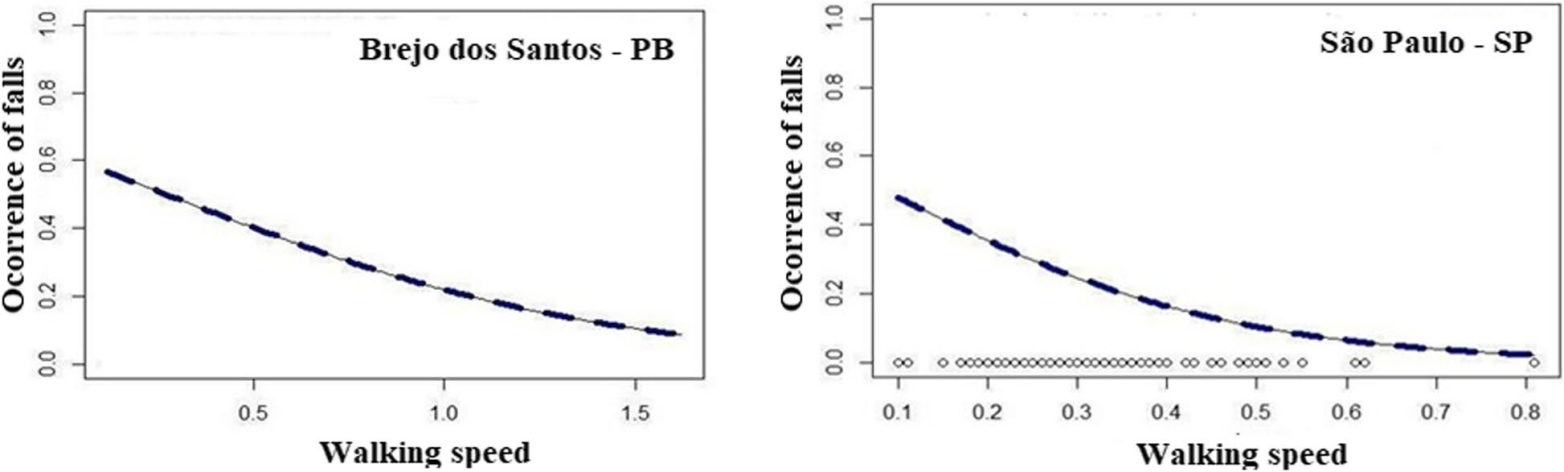
Walking speed and occurrence of falls in longevous population from Brejo dos Santos - PB, Northeastern, and São Paulo - SP, Southeastern, Brazil

Figure 1 shows the logistic regression for walking speed according to the occurrence of falls among the participants of Brejo dos Santos/PB and São Paulo/SP. A significant correlation was verified (*p* = 0.026 and *p* = 0.039, respectively), showing that the lower the walking speed, the higher the predisposition to falls.

Table 2 shows the gross and adjusted values of the factors associated with the occurrence of falls in the rural population of Brejo dos Santos/PB. The gross analysis shows an association between poor balance (OR = 3.46; CI: 1.17–10.23) and moderate balance (OR = 6.29; CI:1.27–31.1) with the occurrence of falls in the previous 12 months. In the adjusted analysis, moderate balance (OR = 5.28; CI: 1.11–25.18) remained associated with the occurrence of falls in the last 12 months.

**Table 2:** Logistic regression model between occurrence of falls, gender, age group, functional capacity and nutritional status. Brejo dos Santos/PB, Brazil, 2017

Variables	Occurrence of falls - Brejo dos Santos						
	n(%)	OR_**gross**_	IC95%	***p***-value	OR_**adjus**_	IC 95%	p-valor
**Demographic aspects**							
Gender							
Female	28(63.6)	1.07	0.50–2.44	0.369			
Male	16(36.4)	1	–				
Age group							
80–89 years	35(79.5)	1	–	0.403			
90 years and over	9(20.5)	1.19	0.49–4.10				
**Functional ability**							
Balance							
Very poor	6(21.4)	3.93	1.00–15.50	0.348	3.31	0.84–13.07	0.088
Poor	11(39.3)	3.46	1.17–10.23	0.107	3.14	0.98–10.02	0.053
Moderate	4(14.3)	6.29	**1.27–31.1**	**0.028**	5.28	**1.11–25.18**	**0.037**
Good	7(25.0)	1	–		1	–	
Lower limb strength							
Very poor	14(50.0)	3.92	0.99–15.51	0.197	2.05	0.44–9.69	0.364
Poor	7(25.0)	2.47	0.54–11.37	0.376	2.05	0.42–10.11	0.376
Moderate	4(14.3)	2.15	0.41–11.20	0.376	1.34	0.22–8.29	0.753
Good	3(10.7)	1	–		1	**–**	
Functional performance							
Unable/very poor	7(25.0)	11	1.1–109.59	0.674			
Low	14(50.0)	4.33	0.54–11.37	0.474			
Moderate	6(21.4)	2.64	0.28–24.60	0.818			
Good	1(3.6)	1	–				
Mobility impaired							
Yes	40(93.0)	1.65	0.43–6.31	0.454			
No	3(7.0)	1	–				
**Nutritional status**							
Body Mass Index							
Underweight	13(35.1)	1.38	0.42–4.52	0.603			
Adequate	15(40.5)	1	–	0.62			
Overweight	9(24.4)	1	0.27–3.75	0.374			
Calf Circumference							
Malnutrition	24(57.1)	0.83	0.31–2.27	0.353			
Eutrophic	18(42.9)	1	–				

Table 3 shows the gross and adjusted values of factors associated with the occurrence of falls among the elderly of this urban population. In the gross analysis, altered mobility (OR = 1.91; CI:0.81–4.49) and very poor functional performance (OR = 3.6; CI:0.70–18.56) were associated with the occurrence of falls. However, in the adjusted analysis, only very poor functional performance (OR = 16.09; CI:1.46–177.06) was associated with the occurrence of falls in the last 12 months.

**Table 3:** Logistic regression model between occurrence of falls, gender, age group, functional capacity and nutritional status. São Paulo/SP, Brazil, 2016

Variables	Occurrence of falls - São Paulo						
	n(%)	OR_**gross**_	IC95%	p-value	OR_**adjus**_	IC 95%	p-valor
**Demographic aspects**							
Sex							
Female	66(73.3)	1.07	0.53–2.13	0.486			
Male	24(26.7)	1	–				
Age group							
80–89 years	63(70.0)	1	–	0.727			
90 years and over	27(30.0)	1.19	0.54–2.60				
**Functional ability**							
Balance							
Very poor	14(21.2)	1.89	0.66–5.39	0.231			
Poor	12(18.2)	1.52	0.52–4.42	0.798			
Moderate	16(24.2)	3	1.21–7.41	0.312			
Good	24(36.4)	1	–				
Lower limb strength							
Very poor	34(68.0)	1.51	0.38–6.08	0.563	0.29	0.03–2.52	0.261
Poor	7(14.0)	1.1	0.22–5.40	0.507	0.33	0.04–3.05	0.332
Moderate	6(12.0)	5.33	0.78–36.33	0.078	4.35	0.52–36.57	0.176
Good	3(6.0)	1	–		1	–	
Functional performance							
Unable/very poor	17(26.2)	3.6	**0.70–18.56**	**0.038**	16.09	**1.46–177.06**	**0.023**
Low	22(33.8)	1.9	0.53–6.76	0.142	7.43	0.9–61.52	0.063
Moderate	22(33.8)	1.54	0.44–5.35	0.122	5.81	0.79–42.68	0.084
Good	4(6.2)	1	–			–	
Mobility impaired							
Yes	78(88.6)	1.91	**0.81–4.49**	**0.03**	2.49	0.94–6.61	0.067
No	10(11.4)	1	–		1	–	
**Nutritional status**							
Body mass index							
Underweight	11(14.9)	0.78	0.26–2.31	0.86			
Adequate	38(51.4))	1	–	0.53			
Overweight	25(33.8)	1.07	0.51–2.28	0.301			
**Calf Circumference**							
Malnutrition	16(18.6)	0.57	0.19–1.67	0.462			
Eutrophic	70(81.4)	1	–				

## Discussion

The present study showed that the prevalence of falls among the seniors in Brejo dos Santos was lower than that found among those living in São Paulo, where a greater proportion of longevous women was observed, corroborating a study that pointed to a greater prevalence of falls among the oldest-old people in the Southeast region than in the Northeast [32]. Significant differences in the female-to-male ratio could explain the findings of these studies, since it is known that women generally experience greater longevity associated with higher rates of disability and poor health than men [33–36], having more risk of falling [32]. The male-female health-survival paradox indicates that men die at younger ages than women, despite better health, because of both biological and environmental differences that include behavioural, cultural, and social factors [33–36]. This difference in life expectancy and mortality indicates that human longevity seems strongly influenced by gender defined as the combination of social and biological factors (e.g. sex hormones, expression of genes, lifestyle or social behaviours) [37–39].

Functional performance is an intrinsic component of the human body affected by the ageing process. Thus, the greatest and most important adversities related to ageing are associated with functional disabilities and dependence; which, in turn, cause loss of skills or difficulty in performing functions and activities related to daily life [40]. The increase in body instability, reduction of flexibility and of reaction time make seniors more prone to falls [41]. This decrease in functional performance is related to the fear of falling that, in turn, may make older people more dependent when performing daily activities, restricting their activities and increasing their propensity to fall [42].

The association of falls with very poor functional performance was observed only in the oldest-old group from São Paulo. On the one hand, it can be explained by female-to-male ratio differences as discussed before. On the other hand, the life habits of the longevous of each region could contribute to increasing the risk of falling. A study carried out by Aires, Paskulin and Morais [43] with 155 longevous individuals belonging to smaller cities or rural areas, showed that they have higher functional performance compared to the seniors from larger cities. This can be explained by the fact that, in their daily lives and in their daily work, the seniors from rural areas or small towns perform activities that make them better able to reach advanced ages with more functionality. According to Barbosa and colleagues [44], the oldest-old in small towns or rural areas have greater social participation than those living in large urban centres and, therefore, may have the greater functional capacity and less physical dependence.

In this study, it was observed that lower gait speeds predisposed both populations to falls, corroborating previous studies [45–47]. In order to maintain body stability and avoid unbalance, older adults make their steps slow and short [45]. Kyrdalen and colleagues [47] observed that both lower and higher walking speed are associated with the occurrence of falls in seniors. High gait speed is associated with falls that happen outdoors and higher levels of physical activity, while low gait speed is associated with falls at home and cognitive and functional decline. In China, for example, a study of 230 oldest-old men aged 80 years and over showed a 4.25 times greater chance of falling among those with lower walking speed [46]. In Norway, in a study of 108 oldest-old people of both sexes, the chance of falling was 3.7 times higher among older people with lower walking speed [47].

Among the oldest-old people living in Brejo dos Santos/PB, the moderate balance was also associated with the occurrence of falls, showing differences with the results of other studies, in which an association between poor or moderate balance and falls was observed [48–50]. Balance alterations may occur due to the human ageing process that compromises the functionality of the central nervous system, which may cause vertigo dizziness and unbalance, which, in turn, predisposes the oldest-old people to falls [51]. The presence of balance alterations in older individuals allows the identification of limitations in the ability to control their movements, as well as the risk of falls [52].

Among the senior residents in São Paulo/SP, the functional performance considered unable or very poor was also associated with the occurrence of falls in the longevous people, corroborating previous studies [48, 53, 54]. In Brazil, a study of 142 older men and women found that individuals with lower functional performance fell 4.16 times more often than those with higher functional performance [54]. A population-based study conducted in Italy with 2710 old people of both sexes aged 60 years or more showed that those with a functional performance score of 6 points or less, considered as poor, were 3.46 times more likely to fall than older people with a score between 10 and 12 points [53].

The literature has pointed out that physical training programmes with different exercise modalities, focusing on balance training, muscle strength, flexibility and endurance, is the most effective and cost-effective fall-prevention strategy in older populations because it can reduce both the number of individuals that fall and the fall rate in fallers [17]. The Brazilian National Policy on Health Promotion recognizes the importance of physical activities and practices involving the body in reducing health risks and improving the quality of life of individuals [55]; however, there are few reports in the literature of government programmes aimed at the older population with an evaluation of their impact [55], as is the case of the “Academy in Town” – a programme implemented in Recife, in Northeast Brazil [56].

In Brejo dos Santos, despite being a small town with 100% coverage of the Family Health Strategy, there was no programme to promote physical activity directed at the older population. A few participants reported doing daily walks. In the city of São Paulo, there are around 46 sports centres with various activities aimed at the older population. In fact, one of the challenges of policies to promote the health of the oldest-old population is to reduce inequalities and asymmetries in the country.

The literature has also shown that changes in home hazards in the oldest-old population may reduce the number of falls and fallers, pointing out that fragility fractures occur indoors [55]. In fall-prevention programmes, a fall decrease of 41% and a reduction in the number of fallers by 24% was verified among the oldest-old people with visual impairment who had removed or changed loose floor mats, painted the edges of steps, reduced glare, installed grab bars and stair rails, removed clutter, and improved lighting [55]. The Brazilian Ministry of Health suggests that the residential environment should be included in an assessment plan for the oldest-old person, as well as other aspects such as the context and mechanism of the falls, the clinical conditions of the oldest-old person, and the medication being used should be investigated. Guidelines aimed at health workers seek to minimise falls and their consequences, such as the adaptation of the environment, considering the home and public places [57].

Brazil is a country with the fifth largest territory in the world, whose regions have relevant social, cultural, ethnic, religious, climatic and biodiversity diversity. In view of these scenarios with discrepant socio-environmental contexts and the multifactorial aspect of falls, the importance of assessing the factors associated with the occurrence of falls in each of the populations is observed, thus enabling differentiated action plans to be outlined. The difference is such that the results of this study show asymmetry among the associated factors observed since the factors associated with the occurrence of falls in the municipality of Brejo dos Santos are of social and economic nature and in São Paulo, on the other hand, the factors are related to biological and behavioural issues.

The limitations of this study concern the design, which limits the interpretations of cause and effect, and the fact that the information was self-reported. Furthermore, due to the fact that the study was developed with populations of longevous people aged 80 years or more, in which there is a high prevalence of cognitive and physical impairment, which made it unfeasible to carry out anthropometric tests and measurements, reducing the final sample of the study. This reduction does not compromise the analysis, considering that it occurred proportionally in both studied populations, urban and rural. Scientific literature has shown that the decrease in functional capacity may be both a causal determinant of falls and a consequence. Therefore, future longitudinal studies may add knowledge to the evidence presented in this study.

## Conclusion

The results pointed out a lower prevalence of falls in longevous people from Brejo dos Santos than in those from São Paulo and differences regarding the associated factors, showing heterogeneity between the two populations. The occurrence of falls was associated with decreased walking speed in the long-lived elderly from both municipalities, with moderate balance in the oldest-old from Brejo dos Santos/PB and with disability for functional performance or very poor functional performance in the oldest-old from São Paulo/SP. These results suggest the need for public policies and effective programmes aimed at preventing falls based on the maintenance or increase of functional capacity, providing and encouraging the regular practice of physical activity, emphasising an acceptable level for the seniors; and providing information for changes of hazards in the home.

## Data Availability

The datasets used and/or analysed during the current study are available from the corresponding author on reasonable request.

## Abbreviations

FC: Functional Capacity
CC: Calf Circumference
BMI: Body Mass Index
SABE: Health, Wellbeing and Ageing Study (*Saúde, Bem-Estar e Envelhecimento*)
